# Comparison of femoral neck system versus cannulated screws for treatment of femoral neck fractures: a systematic review and meta-analysis

**DOI:** 10.1186/s12891-023-06378-x

**Published:** 2023-04-13

**Authors:** Jiabao Jiang, Jialei Chen, Fei Xing, Hao Liu, Zhou Xiang

**Affiliations:** Department of Orthopedics, Orthopedic Research Institute, West China Hospital, Sichuan University, Chengdu, Sichuan Province China

**Keywords:** Femoral neck system, Cannulated screw, Femoral neck fracture, Internal fixation, Systematic review

## Abstract

**Background:**

Recently, some studies on the efficacy of the femoral neck system (FNS) in treating femoral neck fractures (FNFs) have been published. Therefore, a systematic review was performed to clarify the efficacy and safety of FNS versus cannulated screws (CS) for the treatment of FNFs.

**Method:**

The PubMed, EMBASE, and Cochrane databases were systematically searched for studies comparing FNS and CS fixations in FNFs. Intraoperative indicators, postoperative clinical indicators, postoperative complications, and postoperative scores were compared between the implants.

**Results:**

A total of eight studies were included in the study, involving 448 FNFs patients. The results showed that patients in FNS group were significantly lower than the CS group in the number of X-ray exposures (WMD = -10.16; 95% CI, -11.44 to -8.88; *P* < 0.001; I^2^ = 0%), fracture healing time (WMD = -1.54; 95% CI, -2.38 to -0.70; *P* < 0.001; I^2^ = 92%), length of femoral neck shortening (WMD = -2.01; 95% CI, -3.11 to -0.91; *P* < 0.001; I^2^ = 0%), femoral head necrosis (OR = 0.27; 95% CI, 0.08 to 0.83; *P* = 0.02; I^2^ = 0%), implant failure/cutout (OR = 0.28; 95% CI, 0.10 to 0.82; *P* = 0.02; I^2^ = 0%), and Visual Analog Scale Score (WMD = -1.27; 95% CI, -2.51 to -0.04; *P* = 0.04; I^2^ = 91%). And the Harris Score was significantly higher in the FNS group than in the CS group (WMD = 4.15; 95% CI, 1.00 to 7.30; *P* = 0.01; I^2^ = 89%).

**Conclusions:**

Based on this meta-analysis, FNS shows better clinical efficacy and safety in treating FNFs compared to CS. However, due to the limited quality and number of included studies and the high heterogeneity of the meta-analysis; large samples and multicenter RCTs are needed to confirm this conclusion in the future.

**Level of evidence:**

II, Systematic review and Meta-analysis.

**Trial registration:**

PROSPERO CRD42021283646.

## Introduction

In clinical work, femoral neck fractures (FNFs) are a common fracture type. With rising life expectancy throughout the globe, it is estimated that the incidence of FNFs will increase from 1.7 million in 1990 to 6.3 million by 2050 [1]. It is a significant health problem that affects middle-aged and elderly people [2]. The cost of treatment is expensive, causing varying degrees of burden on families and society [3]. Internal fixation is a conventional surgical method for FNFs, especially in nondisplaced FNFs [4]. There are many kinds of internal fixation techniques for FNFs, but there is not one most suitable implant and postoperative complications such as femoral neck shortening, fracture nonunion, and femoral head necrosis are known adverse events [5–8].

Cannulated screws (CS) are currently among the most widely used implants in clinical practice [9, 10]. It has the advantages of minor soft tissue damage, low bleeding, and reliable fixation, but it has poor stability in unstable fracture types [11]. And the total postoperative complication rate is about 46.7% [12]. The Femoral neck system (FNS; DePuy Synthes, Zuchwil, Switzerland) is a new femoral neck internal fixation device launched in 2018. The original intention was to make it minimally invasive and stable. It is more stable than CS in biomechanical studies [11]. Some studies have found that FNS promotes fracture healing and reduces operative time and postoperative complications [13–17]. However, other studies have found that the treatment with FNS did not show significant differences in these aspects compared with CS [18–20].

Therefore, we conducted a meta-analysis to integrate existing data to study the safety and efficacy of FNS and CS in the treatment of FNFs. The study will be evaluated from the following aspects: intraoperative indicators (incision length, blood loss, X-ray exposure, operation time), postoperative clinical indicators (fracture healing time, hospital stay, length of femoral neck shortening), postoperative complications (nonunion/delayed union, femoral head necrosis, implant failure/cutout), and postoperative scores (Visual Analog Scale (VAS) Score, Harris Score). The aim of our study is to provide evidence for orthopedic surgeons to achieve better clinical outcomes when choosing between these two kinds of internal fixation for treating patients with FNFs.

## Material and method

The systematic review was conducted following the PRISMA statement on preferred reporting items on systematic reviews and meta-analyses. The protocol has been registered to PROSPERO (registration number: CRD42021283646).

### Database and searching strategies

We performed a comprehensive, systematic literature search on PubMed, EMBASE and Cochrane. The publication dates were limited from 2018 to February 2022. Search terms included synonyms for FNFs and FNS as follows: ((("Femoral Neck Fractures"[Mesh]) OR ((((Femoral Neck Fractures) OR (Femoral Neck Fracture)) OR (Femur Neck Fractures)) OR (Femur Neck Fracture))) AND (((screw) OR (screws)) OR (implant))) AND ((femoral neck system) OR (fns)). After the search was completed, the relevant literature was searched manually to find potential eligible studies.

### Inclusion criteria

We followed the population/intervention/comparator/outcome/study design (PICOS) principle to develop the inclusion criteria [21]. (1) Population: patients were adults and diagnosed with FNFs. (2) Intervention: patients were treated with FNS. (3) Comparator: patients treated with CS or similar implants [i.e., cannulated compression screw (CCS), inverted cannulated cancellous screw (ICCS), triple screw (TS), and inverted triangle cannulated screw (ITCS)]. (4) Outcomes: studies had at least one of the following clinical outcomes, including intraoperative indicators (incision length, blood loss, X-ray exposure, operation time), postoperative clinical indicators (fracture healing time, hospital stay, length of femoral neck shortening), postoperative complications (nonunion/delayed union, femoral head necrosis, implant failure/cutout), and postoperative scores (VAS Score, Harris Score). (5) Study design: randomized control trails (RCTs), retrospective control studies, and retrospective cohort studies.

### Exclusion criteria

(1) Patients with pathological fractures of the femoral neck, old FNFs, fractures combined with rheumatoid osteoarthritis or hip osteoarthritis, or previous femoral head necrosis. (2) Animal studies. (3) Studies not published in English and Chinese. (4) Studies in which the relevant data could not be extracted, and the original author contacted without response; and (5) biomechanics research and finite element analysis, review articles, expert opinions, case reports, and letters to editors.

### Data extraction

Two reviewers independently extracted the data from all the included studies using a standardized data extraction form to ensure uniform collection. The eligible full-text articles needed to have sufficient data to extract and pool. If the relevant data were not provided in the article, the authors were contacted via email to request the data. The following data were extracted from all eligible studies. Study characteristics: authors, publication year, study design, the sample size of different groups, type/classification of fracture, implants used for internal fixation, and follow-up duration. Clinical outcomes: intraoperative indicators (incision length, blood loss, X-ray exposures, operation time); postoperative clinical indicators (fracture healing time, hospital stay, length of femoral neck shortening); postoperative complications (nonunion/delayed union, femoral head necrosis, implant failure/cutout); and postoperative scores (VAS Score, Harris Score). A third investigator resolved any disagreements through discussion or verification.

### Quality assessment

Non-randomized controlled studies used the MINORS scoring scale [22] to evaluate the following indicators: clearly stated aim; inclusion of consecutive patients; prospective collection of data; endpoints appropriate to the aim of the study; unbiased assessment of the study endpoint; follow-up period appropriate to the aim of the study; loss to follow-up less than 5%; prospective calculation of the study size; adequate control group; contemporary groups; baseline equivalence of groups; and adequate statistical analysis. According to the checklist for MINORS, the highest score for the comparative study was twenty-four. Two independent reviewers conducted a quality assessment and resolved differences through discussion with a third reviewer.

### Statistical analysis

The results of the studies were analyzed using RevMan 5.4 (Copenhagen, The Nordic Cochrane Center, The Cochrane Collaboration, 2020). The weighted mean difference (WMD) with a 95% confidence interval (CI) was used to evaluate continuous outcomes such as blood loss. Odds ratio (OR) with 95% CI was used to assess dichotomous outcomes such as postoperative complications. To measure heterogeneity between studies, we used the I^2^ statistic. A random effect model was applied to combine statistics. Forest plots were used to graphically represent the difference in outcomes of groups of FNS and CS and for all included studies. If *P* was < 0.05, the results were considered statistically significant. The sensitivity analysis was performed to investigate the sources’ heterogeneity and verify the reliability of the results to exclude low-quality studies. We did not evaluate publication bias because when the number of studies was < 10, evaluation of publication bias was not required [23].

## Results

### Included study

**Fig. 1:**
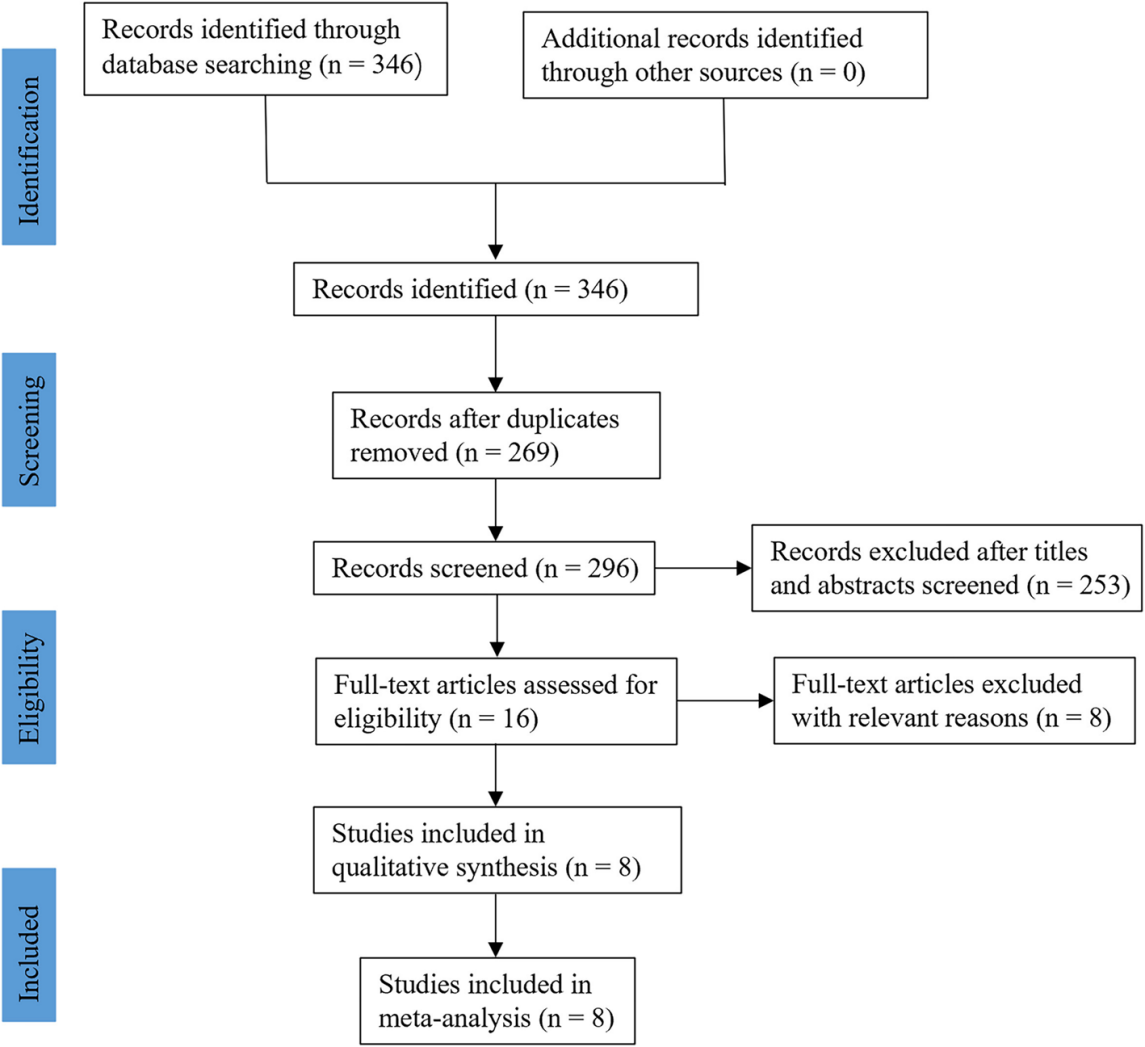
Flow diagram for the identification and selection of studies included in the meta-analysis

We obtained three hundred and forty-six studies through the search strategy. After excluding seventy-seven duplicate records, the remainders were filtered according to the title and abstract, and two hundred and fifty-three were removed. By reading the full text, we excluded eight studies that did not meet the inclusion criteria: four were not in our area of interest, two were ongoing research that has not yet been published, and two were replicated publications. Finally, the systematic review and meta-analysis included eight studies [13–20]. The literature search process is shown in Fig. 1.

### Study characteristics

All included studies were retrospective cohort studies. 448 patients (204 in the FNS group and 244 in the CS group) were enrolled in our study. In these enrolled studies, six studies were conducted in China [13, 15–18, 20], and each one in Switzerland [19] and Japan [14]. The follow-up time ranged from three to twenty-four months. The types of fractures in included studies involved Pauwels I–III and Garden I–IV. The internal fixations used in the included articles compared FNS with CS or their analogs. See Table 1 for more details of the included studies.

**Table 1:** Study characteristics and MINORS scores of included studies

Author (year)	Study design	Sample size		Follow-up duration (mo)	Type/classification of fractures	implants used for internal fixation	MINORS score
		FNS	CS				
Nibe et al. (2021) [14]	RCS	25	5	> 6	Pauwels I–III	FNS and compression hip screw, Hansson Twin Hook, three 6.5-mm cannulated compression screws, Hansson pins	20
Tang et al. (2021) [18]	RCS	47	45	14–24	Pauwels I–III Garden II–IV	FNS and inverted cannulated cancellous screws	20
Hu et al. (2021) [13]	RCS	20	24	> 12	Pauwels I–III Garden I–IV	FNS and Cannulated compression screws	20
Vazquez et al. (2021) [19]	RCS	15	32	NA	Garden I, II Posterior tilt < 20°	FNS and Triple screw construct, dynamic hip screw system	16
Zhou et al. (2021) [17]	RCS	30	30	10–22	Pauwels III	FNS and cannulated screw	20
Yang et al. (2021) [16]	RCS	28	31	3–14	Pauwels III	FNS and inverted triangle cannulated screws	19
Yan et al. (2021) [15]	RCS	24	58	3–18	Pauwels I–III Garden I–IV	FNS and cannulate compression screw	19
Yang et al. (2021) [20]	RCS	15	19	NA	Garden I–IV	FNS and cannulated screw	18

*RCS* Retrospective cohort study, *NA* Not available, *FNS* Femoral neck system, *CS* Cannulated screw

### Quality assessment in the included studies

The mean MINORS score for methodological quality assessment was 19/24 (range from 16 to 20) (Table 1). All studies received two points deduction for their retrospective study design, four studies [15, 16, 19, 20] lost points in the follow-up period (two studies [19, 20] did not mention the follow-up period, and two [15, 16] reported an inadequate follow-up period), and one study [19] lost point because of the baseline data inequality.

### Meta-analysis of intraoperative indicators

**Fig. 2:**
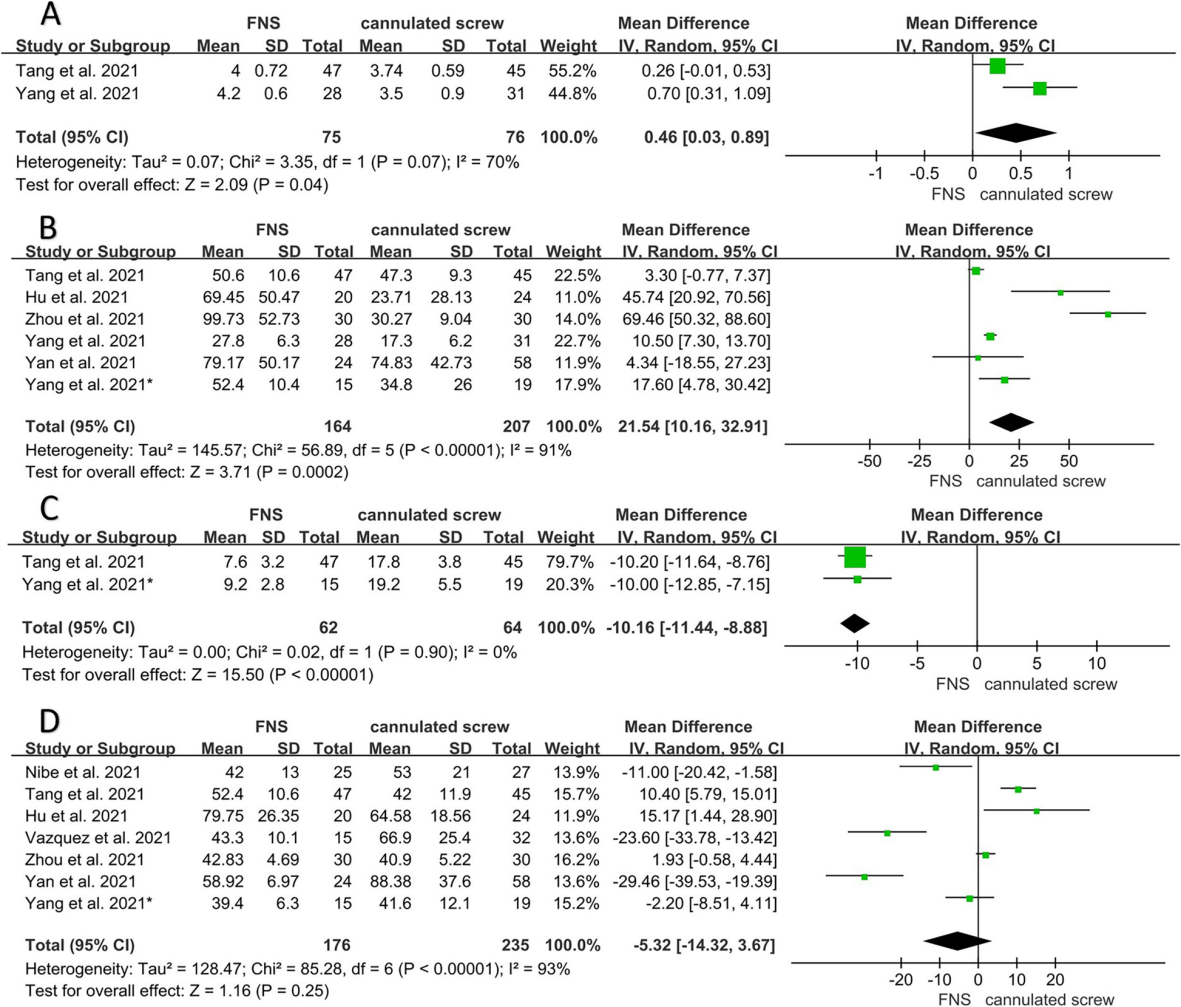
Forest plot of the intraoperative indicators between the FNS and cannulated screw groups. **A** Incision length. **B** Blood loss. **C** X-ray exposures. **D** Operation time

The results of each indicator during the operation are shown in Table 2. Two studies reported the length of the surgical incision [16, 18]. The incision length of the CS group was significantly smaller than in the FNS group (WMD = 0.46; 95% CI, 0.03 to 0.89; *P* = 0.04) (Fig. 2A). Six studies provided data on intraoperative blood loss [13, 15–18, 20]. The results showed that blood loss during the operation was significantly less in the CS group than in the FNS group (WMD = 21.54; 95% CI, 10.16 to 32.91; *P* < 0.001) (Fig. 2B). Two studies compared the number of intraoperative X-ray exposures [18, 20]. The results showed that the number of fluoroscopies in the FNS group were significantly less than in the CS group (WMD = -10.16; 95% CI, -11.44 to -8.88; *P* < 0.001) (Fig. 2C). Eight studies [13–20] all reported the operation time, and the data of one study [16] was excluded due to the use of the median method for statistics. There was no difference in operation time between the two groups (WMD = -5.32; 95% CI, -14.32 to 3.67; *P* = 0.25) (Fig. 2D).

**Table 2:** Weighted mean differences or odd ratios of outcomes following each analysis comparing FNS to CS

Subgroup and Outcomes	No. of studies	Sample size		WMD or OR (95% CI)	I^2^, %	*P* value
		FNS	CS			
**Intraoperative indicators**						
Incision length (cm)	2	75	76	WMD = 0.46 (0.03, 0.89)	70	0.04
Blood loss (ml)	6	164	207	WMD = 21.54 (10.16, 32.91)	91	< 0.001
X-ray exposures (n)	2	62	64	WMD = -10.16 (-11.44, -8.88)	0	< 0.001
Operation time (min)	7	176	235	WMD = -5.32 (-14.32, 3.67)	93	0.25
**Postoperative clinical indicators**						
Fracture healing time (mo)	4	119	158	WMD = -1.54 (-2.38, -0.70)	92	< 0.001
Hospital stay (day)	4	120	138	WMD = 0.17 (-0.31, 0.65)	52	0.49
Length of femoral neck shortening (mm)	3	63	87	WMD = -2.01 (-3.11, -0.91)	0	< 0.001
**Postoperative complications**						
Nonunion/delayed union (n)	6	161	181	OR = 0.43 (0.16, 1.13)	0	0.09
Femoral head necrosis (n)	6	174	193	OR = 0.27 (0.08, 0.83)	0	0.02
Implant failure/cutout (n)	4	125	130	OR = 0.28 (0.10, 0.82)	0	0.02
**Postoperative scores**						
Visual Analog Scale Score	2	54	88	WMD = -1.27 (-2.51, -0.04)	91	0.04
Harris Score	6	164	207	WMD = 4.15 (1.00, 7.30)	89	0.01

*WMD* Weighted mean difference, *OR* Odd ratio, *FNS* Femoral neck system, *CS* Cannulated screw

### Meta-analysis of postoperative clinical indicators

**Fig. 3:**
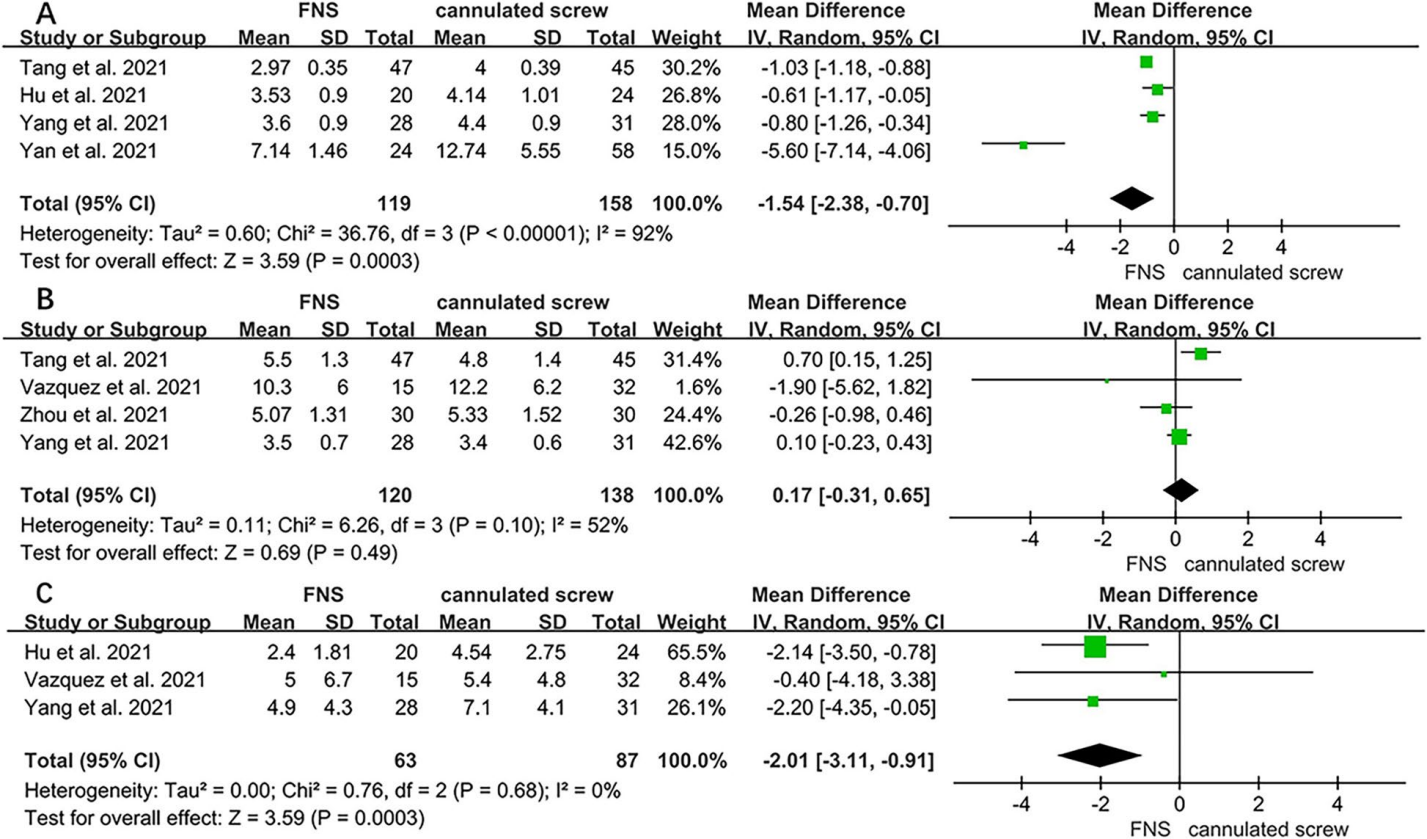
Forest plot of the postoperative clinical indicators between the FNS and cannulated screw groups. **A** Fracture healing time. **B** Hospital stay. **C** length of femoral neck shortening

The postoperative clinical indicators are shown in Table 2. Four studies [13, 15, 16, 18] reported fracture healing time. The FNS group was significantly better than the CS group in terms of fracture healing time (WMD = -1.54; 95% CI, -2.38 to -0.70; *P* < 0.001) (Fig. 3A). The length of hospital stay was extracted from four studies [15, 17–19]. There was no significant difference between the two groups (WMD = 0.17; 95% CI, -0.31 to 0.65; *P* = 0.49) (Fig. 3B). Three studies [13, 16, 19] provided data on the length of femoral neck shortening. Compared with CS, FNS can better prevent femoral neck shortening (WMD = -2.01; 95% CI, -3.11 to -0.91; *P* < 0.001) (Fig. 3C).

### Meta-analysis of postoperative complications

**Fig. 4:**
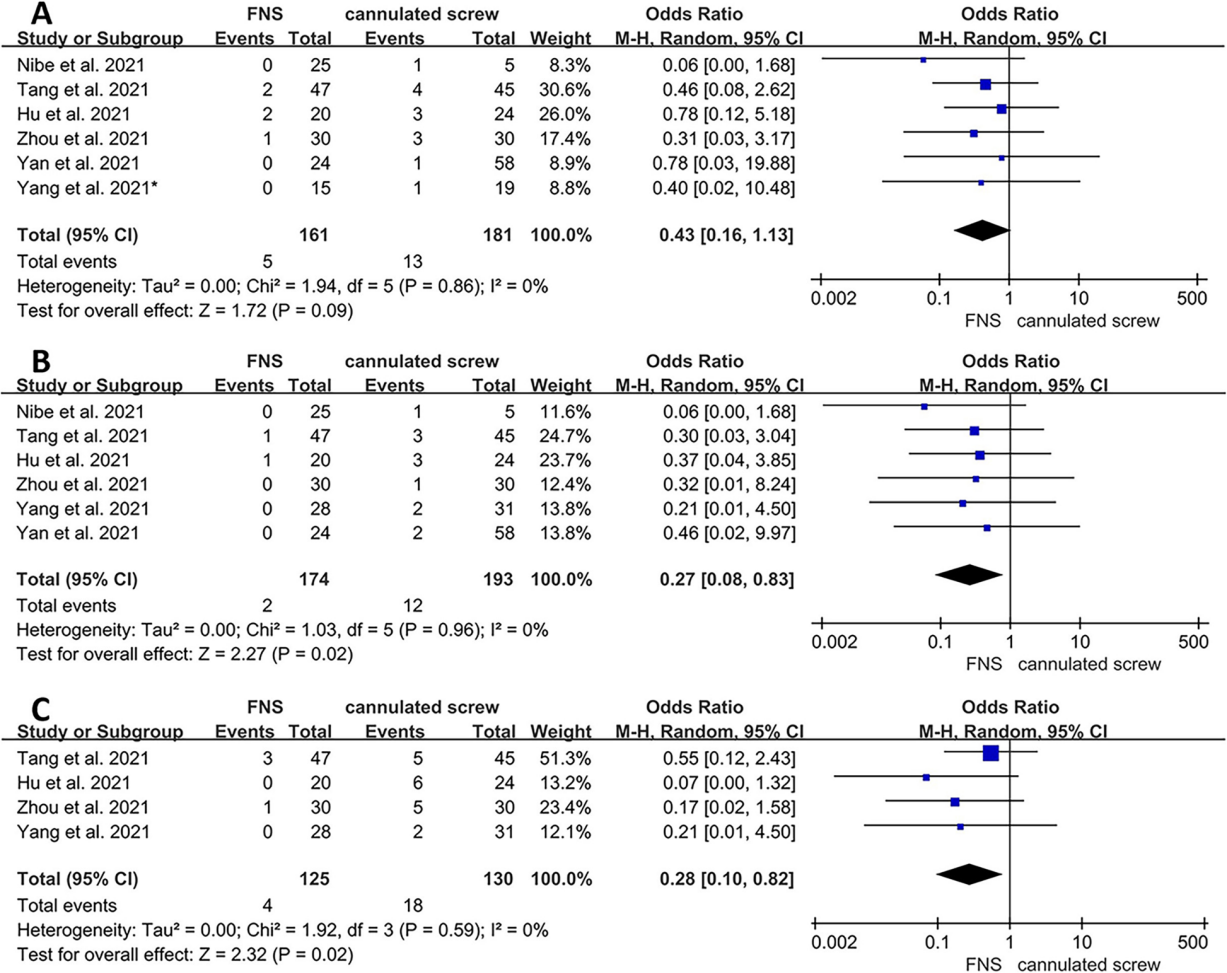
Forest plot of the postoperative complications between the FNS and cannulated screw groups. **A** Nonunion/delayed union. **B** Femoral head necrosis. **C** Implant failure/cutout

The detailed results of postoperative complications are shown in Table 2. Six studies [13–15, 17, 18, 20] included patients with nonunion/delayed union after internal fixation. The results showed no significant difference between the two groups (OR = 0.43; 95% CI, 0.16 to 1.13; *P* = 0.09) (Fig. 4A). Six studies [13–18] reported the occurrence of postoperative femoral head necrosis. The incidence of femoral head necrosis in the FNS group was significantly lower than in the CS group (OR = 0.27; 95% CI, 0.08 to 0.83; *P* = 0.02) (Fig. 4B). Four studies [13, 16–18] reported the occurrence of implant failure/cutout. The incidence of implant failure/cutout was significantly lower in the FNS group than in the CS group (OR = 0.28; 95% CI, 0.10 to 0.82; *P* = 0.02) (Fig. 4C).

### Meta-analysis of postoperative scores

**Fig. 5:**
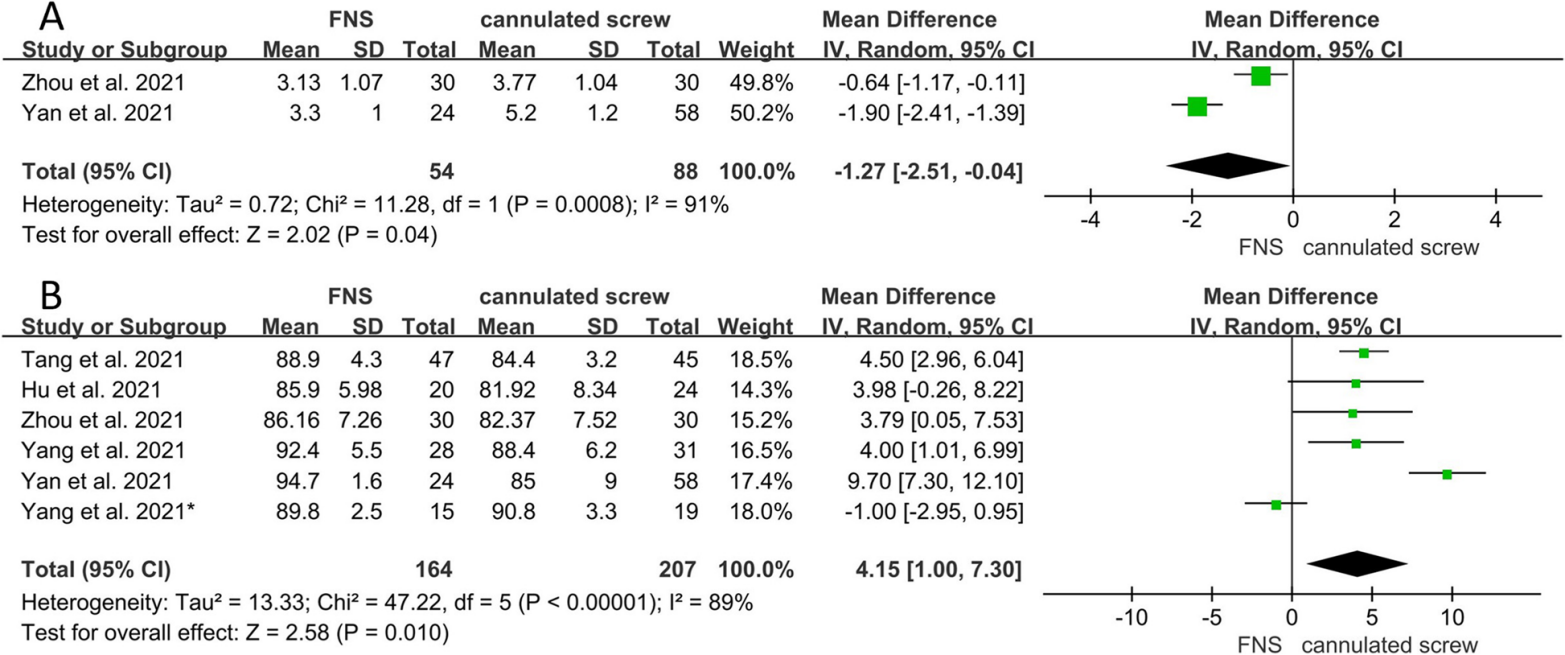
Forest plot of the postoperative scores between the FNS and cannulated screw groups. **A** Visual Analog Scale Score. **B** Harris Score

The detailed results of the postoperative scores are shown in Table 2. Two studies provided data on postoperative VAS Score [15, 17]. The VAS Score was significantly lower in the FNS group than in the CS group (WMD = -1.27; 95% CI, -2.51 to -0.04; *P* = 0.04) (Fig. 5A). Six studies [13, 15–18, 20] reported the Harris Score. The FNS group was significantly better than the CS group in the Harris Score (WMD = 4.15; 95% CI, 1.00 to 7.30; *P* = 0.01) (Fig. 5B).

### Sensitivity analysis

A sensitivity analysis was performed by individually removing each study to determine whether the pooled results changed. When the study by Yan et al. [15] was removed, the heterogeneity of fracture healing time was reduced (*P* < 0.001, I^2^ = 25%). And when Tang et al.’s study [18] was removed, the length of hospital stays without additional heterogeneity (*P* = 0.88, I^2^ = 0%). The pooled results of blood loss, operation time, and Harris Score were stable.

## Discussion

From the perspective of biomechanical studies compared with the CS, the FNS has a higher angle stability in the unstable femoral neck fracture model, and has a strong ability to resist varus deformity [11, 24]. However, due to the limited clinical application time, only a few studies have compared the therapeutic effects of the two types of implants, and the sample size in different studies was small. Therefore, we have formulated comprehensive and rigorous inclusion and exclusion criteria based on published studies to evaluate the safety and efficacy of FNS and CS in the treatment of FNFs. The results showed that FNS is more effective than CS in decreasing the number of X-ray exposures, fracture healing time, length of femoral neck shortening, femoral head necrosis, implant failure/cutout and postoperative VAS Score. It can also significantly improve the postoperative Harris Score.

### Intraoperative indicators

The CS group could significantly reduce blood loss compared with the FNS group in terms of intraoperative indicators. Because when using CS to treat FNFs, only a small incision is needed to implant the screws. When FNS is used, a longitudinal incision is required to implant the FNS device due to its structural characteristics. CS should be more advantageous for soft tissue injuries and intraoperative blood loss. Regardless of the CCS, ICCS, TS, or ITCS, to maximize the stability of the structure and accelerate fracture healing, they all need a triangular distribution, and the screws should be implanted as parallel as possible in anteroposterior X-rays [7, 25]. But there is no correlation between the screws, and the position of the screws in the femoral neck needs to be adjusted multiple times. Therefore, various fluoroscopies cannot be avoided during the operation to determine the position of screws. The design of the FNS device simplifies the surgical procedure. It only needs to insert a 130° guide and a central positioning guide pin to complete the implantation of the internal fixation, which can effectively reduce the number of intraoperative fluoroscopies [18]. It is generally believed that repeated intraoperative fluoroscopies will prolong the operation time when CS is used, but we found no difference in the operation time between the two types of implants. The possible reason is the insufficient time of FNS devices for clinical application, and orthopedic surgeons have not fully mastered the surgical skills, which leads to the prolonged operation time. However, most studies did not report the seniority of surgeons. Only one indicated that the operation was performed by four residents under the supervision of a consultant and by seven surgeons [19].To explore the potential sources of heterogeneity, we performed a sensitivity analysis. The results were consistent with previous results.

### Postoperative clinical indicators

In the postoperative clinical indicators, the FNS group was significantly better than the CS group in terms of femoral neck shortening and fracture healing time, and there was no difference in hospitalization time between the implants. One of the characteristics of FNS is dynamic compression [11]. The pre-collapsed insertion allows the anti-rotation screw and bolt slide in the maximum 20 mm packaging to meet femoral neck shortening during fracture healing. Because within a certain range, the impaction of the fracture gap can accelerate the healing of the fracture [26]. However, it is generally considered that shortening > 10 mm is severe femoral neck shortening, which is detrimental to fracture healing and postoperative function [27]. The biomechanical properties of multiple screws cannot fully resist the high shear force around the hip, and severe shortening is prone to occur after surgery [28]. Zlowodzki et al. [29] found that the shortening rates after fracture fixation with multiple cancellous screws of non-displaced and displaced femoral neck fractures were 31% and 27%, respectively. Angular stable devices, including dynamic hip screw and FNS, have advantages in resisting high shear forces and femoral neck shortening [11]. Our systematic review showed that Compared with CS, FNS can effectively prevent femoral neck shortening and accelerate fracture healing time, which is consistent with the results of previous in *vitro* studies [11]. The high heterogeneity of the results may be due to the different types of fractures included in the studies, but the limitation of the number of existing studies, we cannot perform subgroup analysis. More research should be conducted in the future.

### Postoperative complications

In terms of postoperative complications, we observed a significant reduction in femoral head necrosis and internal fixation failure/cut-out in the FNS group. Although the nonunion/delayed union of the fracture of the two types of implants was not significantly different, the incidence in the FNS group was lower. This is a meaningful discovery for clinical treatment. The most commonly used internal fixation device for treating FNFs in clinics is the CS, which has the advantage of minimal intraoperative soft tissue damage and compression fixation of the fracture site. However, its resistance to shear and rotational stresses is insufficient, and even with good intraoperative repositioning, postoperative complications such as nonunion of the fracture, screw excision, and femoral head necrosis are prone to occur, especially in unstable FNFs [30]. These complications are also the main cause of post-operative reoperation, bringing huge risks to the patient's quality of life and financial burden [31]. There are also adjuvant therapies combined with surgery that can decrease the rate of femoral head necrosis, such as platelet-rich plasma or stem cells [32–34]. However, there are no reports of FNS in combination with other adjuvant therapies. Our systematic review results showed that FNS effectively reduces the incidence of postoperative complications, which is beneficial for clinical application and demonstrates the safety and efficacy of this new internal fixation device.

### Postoperative scores

The VAS Score is an important indicator to assess the degree of pain in patients. A lower score is associated with lower postoperative pain. Harris Score is the most frequently used scale to evaluate the postoperative function of the hip joint. It mainly evaluates four aspects: pain, daily activities, deformity, and range of motion. The higher the score, the better the individual's postoperative recovery [35]. The results of our systematic review showed that the FNS group could significantly improve the Harris Score compared to the CS group. We believe that the FNS group had a better score because it can effectively prevent postoperative femoral neck shortening. Many studies have found that shortening of the femoral neck leads to inferior hip function. The more severe the shortening, the worse the function [27, 29].

### Limitations

This study had the following limitations: (1) all the included studies were retrospective and observational, there was a risk of selection bias, and systemic and random errors were prone to occur; (2) the follow-up duration of the included studies is relatively short, and some postoperative complications may not occur; and (3) the number of included studies is small, and the level of evidence is not high due to the lack of RCTs, more high-quality researches are needed in the future to improve the reliability of the results.

## Conclusion

This systematic review indicates that FNS is a safe and effective internal fixation device. However, due to the limited quality and number of included studies and the high heterogeneity of the meta-analysis; large samples and multicenter RCTs are needed to confirm this conclusion in the future.

## Data Availability

All data generated or analyzed during this study are included in this published article.

## Abbreviations

FNS: Femoral Neck System
FNFs: Femoral Neck Fractures
CS: Cannulated Screws
VAS: Visual Analog Scale
PRISMA: Pre-ferred Items Reporting for Systematic Reviews and Meta-Analysis
PROSPERO: International Prospective Register of Systematic Reviews
PICOS: Population/Intervention/Comparator/Outcome/Study Design
RCTs: Randomized Control Trails
CCS: Cannulated Compression Screw
ICCS: Inverted Cannulated Cancellous Screw
TS: Triple Screw
ITCS: Inverted Triangle Cannulated Screw
MINORS: Methodological Index for Non-randomized Studies
WMD: Weighted Mean Difference
CI: Confidence Interval
OR: Odds Ratio
